# 3D ICE-Guided T-TEER Performed by a Single Operator With Limited Imaging Support and Moderate Sedation

**DOI:** 10.1016/j.jscai.2025.103603

**Published:** 2025-03-26

**Authors:** Scott J. Hoffman, Kari J. Remmick

**Affiliations:** Division of Cardiology, Park Nicollet Methodist Hospital, St Louis Park, Minnesota

**Keywords:** 3-Dimensional intracardiac echocardiography, moderate sedation, transcatheter edge-to-edge repair, TriClip

## Case presentation

An 85-year-old woman with torrential (5+) functional tricuspid regurgitation (TR) by transthoracic echocardiography, in the setting of permanent atrial fibrillation, was evaluated for transcatheter tricuspid edge-to-edge repair (T-TEER) after being recurrently hospitalized for acute right-sided heart failure despite optimal guideline-directed medical therapy (Supplemental Video 1A). Preprocedural transesophageal echocardiography (TEE) imaging provided suboptimal tricuspid valve (TV) windows; thus, after a Heart Team discussion, the patient elected to proceed with T-TEER using 3-dimensional intracardiac echocardiography (3D ICE) for imaging guidance. Due to the semiurgent need for the intervention and scheduling constraints for multidisciplinary team members, the procedure was performed by a single structural interventionalist, with imaging support provided by a sonographer who managed the ultrasound console.

**Figure 1:**
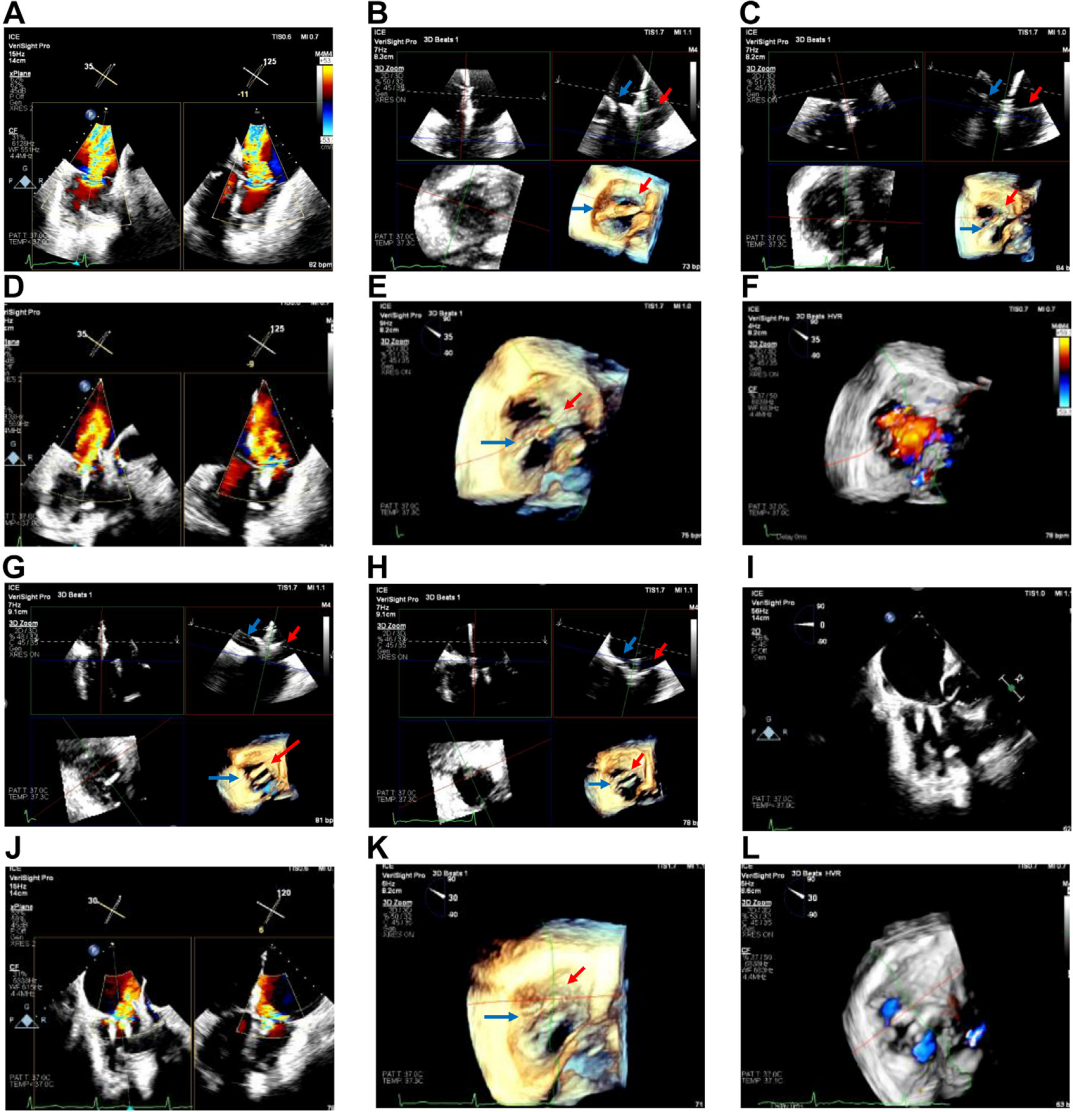
**Transcatheter tricuspid edge-to-edge repair with 3-dimensional intracardiac echocardiography guidance.** (A) Torrential (5+) TR, by 2D x-plane color imaging, originating from a gap between the posterior (blue arrow) and septal (red arrow) tricuspid valve leaflets. (B) Mid-leaflet alignment of XTW TriClip, and (C) grasp of posterior and septal leaflets, in MPR. (D) Severe (3+) residual TR, by 2D x-plane color imaging and MPR (E) with, and (F) without color imaging. (G) Commissural alignment of second XTW TriClip, and (H) grasp of posterior and septal leaflets, in MPR. (I) Deployed XTW devices in 2D, with moderate (2+) residual TR, by (J) 2D x-plane color imaging and MPR (K) with, and (L) without color imaging. 2D, 2-dimensional; MPR, multiplanar reconstruction; TR, tricuspid regurgitation.

Moderate sedation was administered. Bilateral femoral venous access was obtained. A VeriSight Pro 3D ICE catheter (Philips) was introduced into the right atrium, and a pre-TEER assessment was performed, revealing mean TV gradient of 1 mm Hg and torrential (5+) TR primarily originating from a posteroseptal coaptation gap; thus, an XTW TriClip (Abbott) was selected (Figure 1A; Supplemental Video 1B). A TriClip guide was inserted, followed by the XTW device.

Multiplanar reconstruction (MPR) imaging was used to guide the clip toward the mid-aspect of the posteroseptal coaptation gap, advance it into the right ventricle, and grasp the TV leaflets (Figure 1B, C; Supplemental Video 2). After ICE evaluation, the clip was released, leaving severe (3+) TR in the posteroseptal commissure (Figure 1D-F). Thus, a second XTW device was deployed under MPR guidance, resulting in moderate (2+) residual TR and mean TV gradient of 2 mm Hg (Figure 1G-L; Supplemental Videos 3, 4A). Total procedure time was 108 minutes. The patient was discharged the next day, after transthoracic echocardiography confirmed stable TriClip findings (Supplemental Video 4B).

## Discussion

To our knowledge, this is the first report of 3D ICE-guided T-TEER performed under moderate sedation by a single operator without echocardiographer support. Previously, 3D ICE has been used as an adjunctive imaging modality during TEER procedures if TEE imaging windows are suboptimal, or as the sole imaging modality when TEE or general anesthesia is contraindicated,^1^^,^^2^ usually with a combination of sonographers, echocardiographers, and industry ICE specialists who perform image acquisition and interpretation.

Given the challenges of TV imaging from the esophagus, 3D ICE is increasingly being used to support T-TEER because of its ability to provide high-quality, largely unobstructed near-field and MPR images from the right atrium. However, multimodality imaging with TEE and 3D ICE is not sustainable because it often increases procedural staffing demands and adds considerable expense. Furthermore, it does not eliminate general anesthesia and intubation, which exclude certain patients from undergoing TEER, increase the risk of complications, and delay recovery times.^3^^,^^4^

Conversely, despite an initial steep learning curve, 3D ICE-guided T-TEER performed under moderate sedation by a sole operator with focused imaging support represents a true minimalist approach that could expand treatment to more patients, lower procedural risk, streamline workflows, optimize support staff utilization, and reduce cost, similar to other percutaneous interventions.

## Supplementary material

Supplementary Videos

## Footnotes

To access the supplementary material accompanying this article, visit the online version of the *Journal of the Society for Cardiovascular Angiography & Interventions* at 10.1016/j.jscai.2025.103603.

## References

[1] Singh G.D., Aman E., Pham T., Rogers J.H., Astina K., Smith T.W.R. (2022). vICE-guided T-TEER in a patient without TEE windows. JACC Cardiovasc Interv.

[2] Hoffman S.J., Hari P.K., Sarcia P.J., Reiff C.J., Smith A.J., Sen S. (2022). Mitral valve transcatheter edge-to-edge repair performed exclusively with 3-dimensional intracardiac echocardiography and moderate sedation. J Soc Cardiovasc Aniogr Interv.

[3] Hasnie A.A., Parcha V., Hawi R. (2023). Complications associated with transesophageal echocardiography in transcatheter structural cardiac interventions. J Am Soc Echocardiogr.

[4] Butala N.M., Chung M., Secemsky E.A. (2020). Conscious sedation versus general anesthesia for transcatheter aortic valve replacement: variation in practice and outcomes. JACC Cardiovasc Interv.

