# Supplementary material for: 3D ICE-Guided T-TEER Performed by a Single Operator With Limited Imaging Support and Moderate Sedation
Source: J Soc Cardiovasc Angiogr Interv. 2025 Mar 26;4(6):103603. doi: 10.1016/j.jscai.2025.103603 (PMC12230489; doi:10.1016/j.jscai.2025.103603)
Supplement: Supplementary Videos [file mmc1.pptx]

## Slide 1
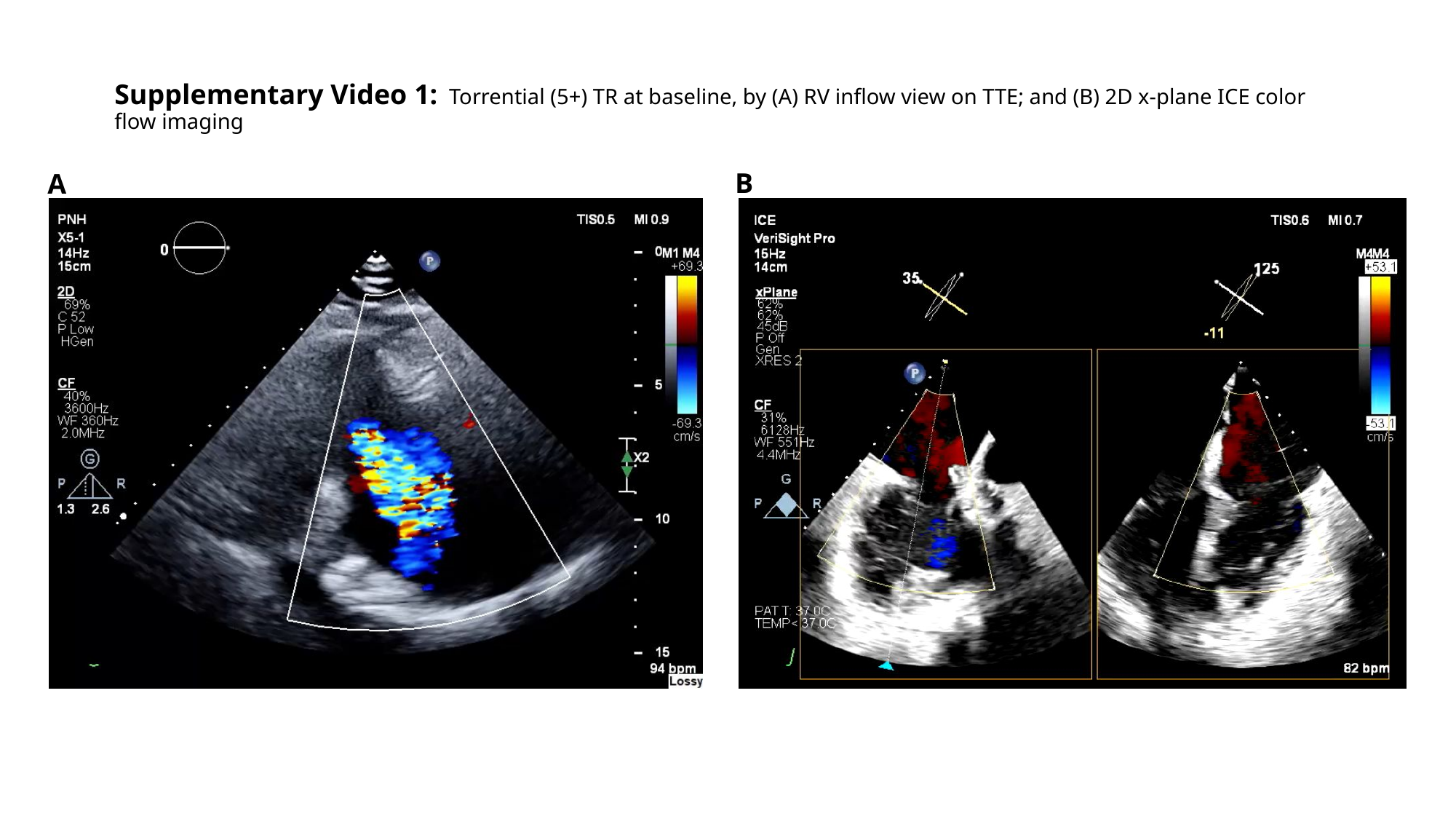

# Supplementary Video 1: Torrential (5+) TR at baseline, by (A) RV inflow view on TTE; and (B) 2D x-plane ICE color flow imaging
B
A

## Slide 2
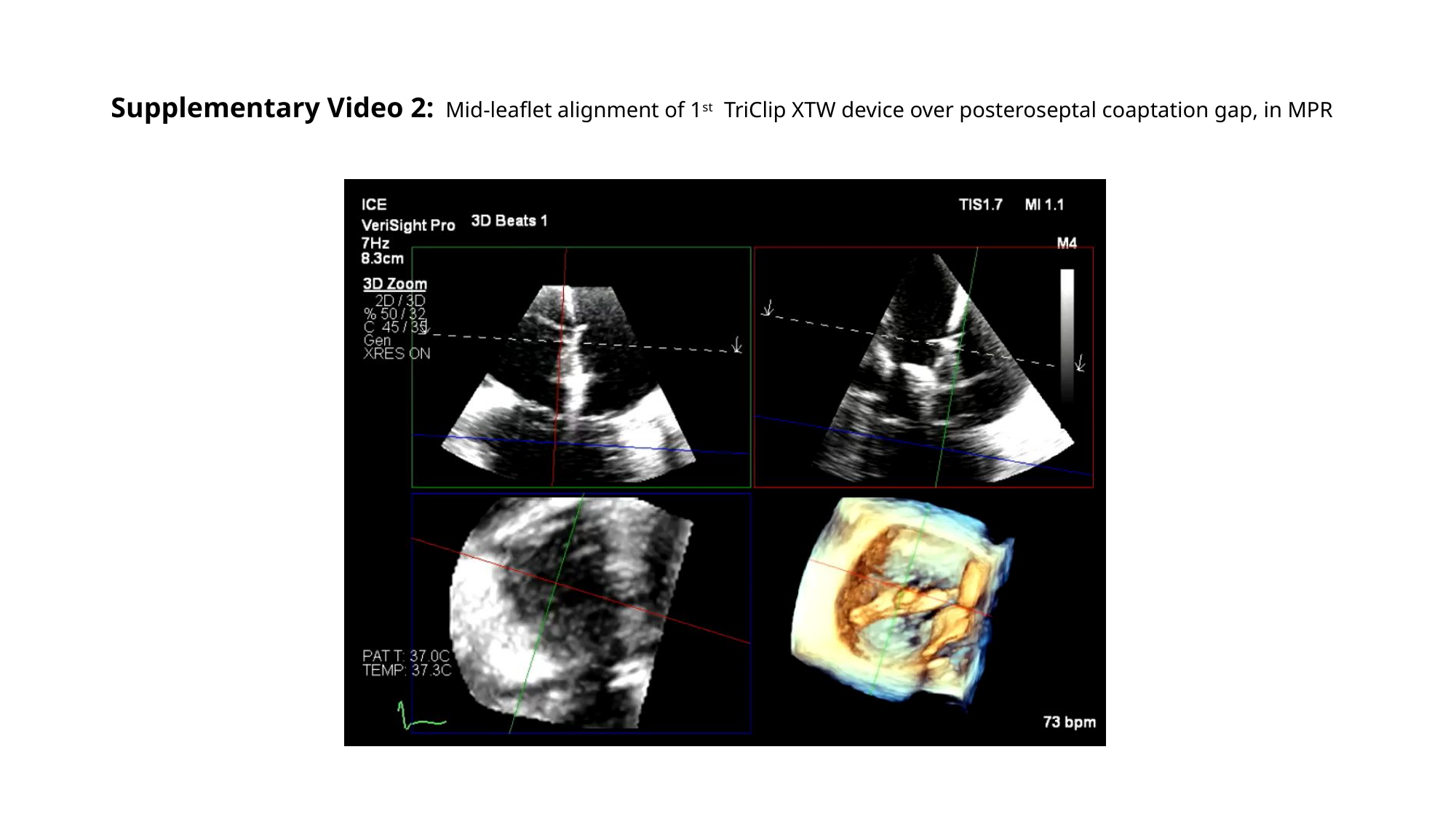

# Supplementary Video 2: Mid-leaflet alignment of 1st TriClip XTW device over posteroseptal coaptation gap, in MPR

## Slide 3
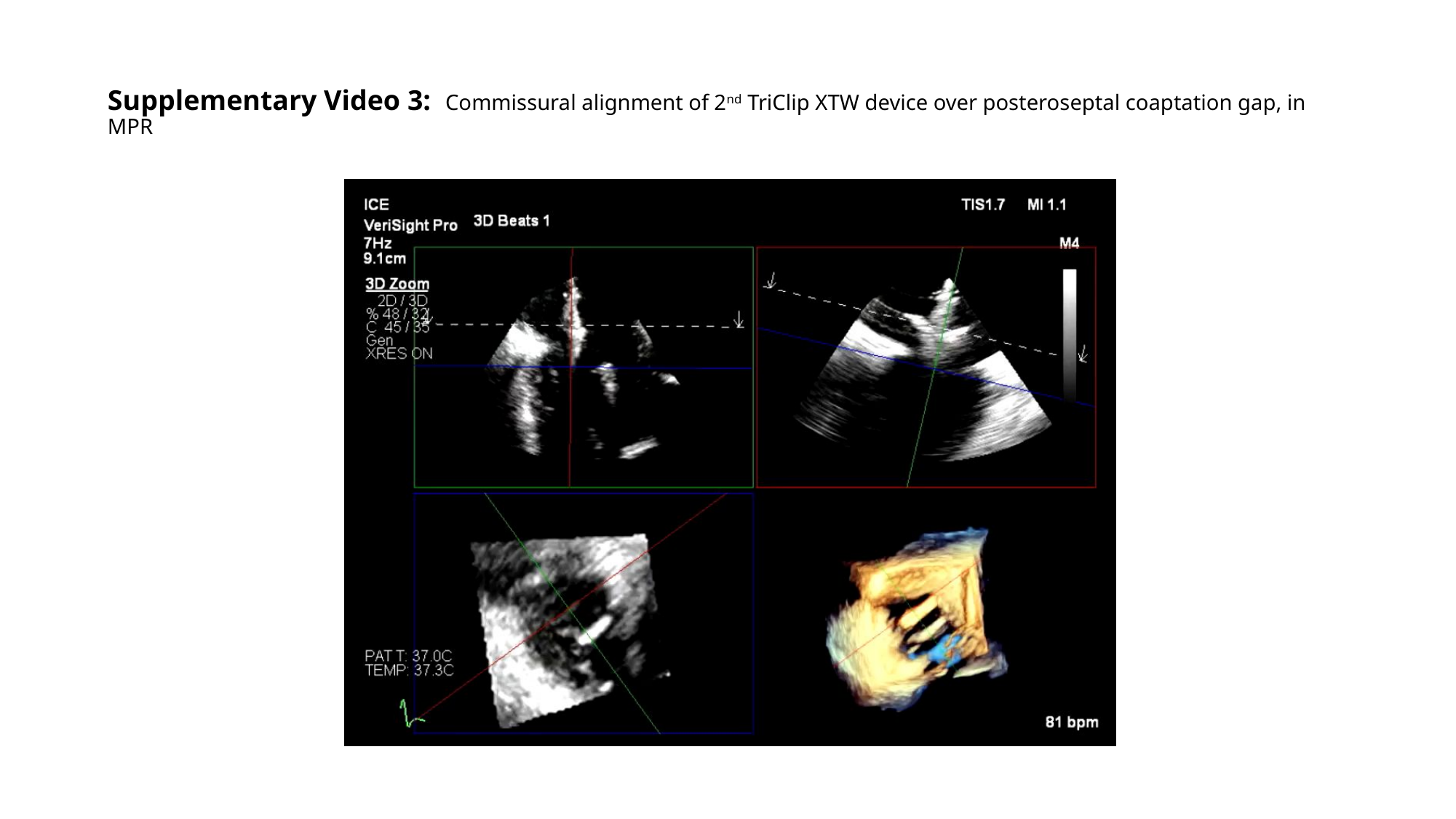

# Supplementary Video 3: Commissural alignment of 2nd TriClip XTW device over posteroseptal coaptation gap, in MPR

## Slide 4
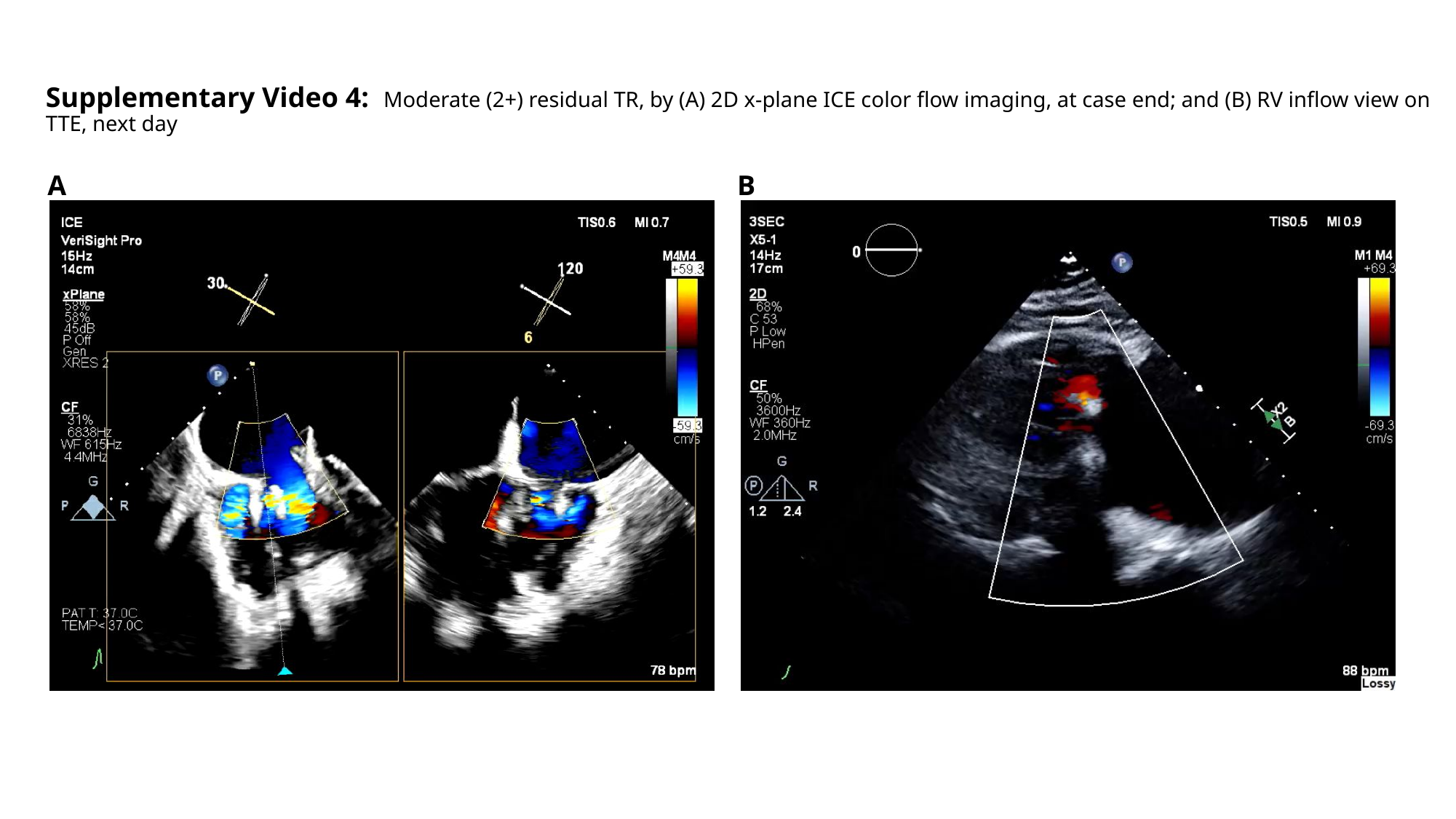

# Supplementary Video 4: Moderate (2+) residual TR, by (A) 2D x-plane ICE color flow imaging, at case end; and (B) RV inflow view on TTE, next day
B
A
